# Longitudinal Changes of Cytokines and Appetite in Older Hospitalized Patients

**DOI:** 10.3390/nu13082508

**Published:** 2021-07-22

**Authors:** Maryam Pourhassan, Nina Babel, Lars Sieske, Timm Henning Westhoff, Rainer Wirth

**Affiliations:** 1 Department of Geriatric Medicine, Marien Hospital Herne, Ruhr-Universität Bochum, Hölkeskampring 40D, 44625 Herne, Germany; lsieske@googlemail.com (L.S.); Rainer.Wirth@elisabethgruppe.de (R.W.); 2 Medical Department I, General Internal Medicine, Marien Hospital Herne, Ruhr-Universität Bochum, 44625 Herne, Germany; Nina.Babel@elisabethgruppe.de (N.B.); Timm.Westhoff@elisabethgruppe.de (T.H.W.)

**Keywords:** appetite, cytokines, inflammation, interleukin-18, older persons

## Abstract

There are few data on the longitudinal association of cytokine and appetite among older hospitalized patients. We aimed to investigate the impact of the changes of inflammatory cytokines on appetite in older hospitalized patients. A total of 191 patients (mean age 81.3 ± 6.6 years, 64% women) participated in this prospective longitudinal observational study. Appetite was evaluated using the Edmonton Symptom Assessment System on admission and after seven days. Serum cytokines such as IL-1β, IL-6, IL-8, IL-10, IL-12p70, IL-17, IL-18, IL-23 and IL-33, IFN-α2, IFN-γ, TNF-α and MCP-1 were measured both times. No significant differences in the mean serum levels of all the cytokines could be detected overtime in relation to appetite changes, except for IL-18. Appetite significantly deteriorated overtime in patients with increasing IL-18 levels and improved in those without significant changes in IL-18 levels. In a stepwise regression analysis, changes of IL-18 levels were the major independent predictor for the changes of patients’ appetite and explained 4% of the variance, whereas other cytokines and variables, such as age, sex, infection and disease, did not show any impact on appetite changes. We conclude that IL-18 seems to exert a significant impact on appetite in acutely ill older hospitalized patients and should, therefore, be considered as a potential target in the diagnosis, prevention and treatment of malnutrition.

## 1. Introduction

The process of aging is associated with physical, physiological and psychological alteration that may negatively affect appetite and food intake [1]. This condition has been identified as the anorexia of aging and it may potentially lead to the development of malnutrition [2,3,4]. Malnutrition is a frequent complication in older persons. Its etiology in this group is mostly multifactorial and not entirely understood [5]. The pathophysiology of malnutrition may be mediated by low nutritional intake, increased nutritional demands and decreased bioavailability of nutrients [5]. However, low nutritional intake seems to be the key factor in older persons. One of the major risk factors for low nutritional intake is poor appetite with a prevalence of 15% in community-dwelling older persons [6] and 33% in older hospitalized patients [7,8]. Poor appetite and impaired food consumption are often associated with chronic and/or acute illness, which may simultaneously increase energy requirements and accelerate the development of malnutrition in older adults [5,9].

Inflammation and malnutrition tend to occur simultaneously in older adults, which led to the term malnutrition-inflammation-complex syndrome [10,11]. Inflammation exerts an influence on appetite and, thus, lowers food intake [12], which is not yet sufficiently understood. In response to inflammatory conditions, a number of cytokines are synthesized and released. In addition to their biological effects to cope with infection and injury, it has been shown that the accumulation of cytokines potentially diminishes appetite and changes feeding behavior by interacting with the hypothalamus, which is the center for energy homeostasis and appetite [13,14]. Nevertheless, which cytokines have the primary impact on human appetite is unknown.

The association of inflammation expressed by increased levels of cytokines with appetite and food intake has been already confirmed in cross-sectional studies among patients with advanced cancer, renal disease, Alzheimer’s disease, depression, infectious diseases and acute disease in general [15,16,17,18]. However, there are relatively few longitudinal studies testing such associations.

To the best of our knowledge, there are no data on the association of cytokine changes with appetite changes among older persons. It is understood that cytokines may lead to poor appetite, but the extent to which the changes of cytokines can alter appetite and food intake and which cytokines play a major role are not known. Although, causality cannot be proven using observational research, simultaneous longitudinal changes and a dose–response relationship would substantiate a probable causality. That is why we conducted this longitudinal study. We investigated the impact of changes of the main inflammatory cytokines on appetite and food-intake in older hospitalized patients, in order to highlight the pathophysiology of the inflammation-associated loss of appetite.

## 2. Subjects and Methods

### 2.1. Study Design and Subjects

Two hundred patients, who were consecutively admitted to the geriatric department of our university hospital in the period from September 2017 to November 2018 participated in this prospective longitudinal observational study. A detailed description of the study population and methods has been reported in more detail elsewhere [8,19,20]. Nine patients were excluded due to missing values of serum cytokines. Therefore, one hundred and ninety-one older patients were included in the study (122 females). The study population is a typical mixture of geriatric multi-comorbidity including chronic, inflammatory diseases. Indeed, we cannot exclude that some of the patients display chronic inflammation; however, the majority of the population was admitted to hospital due to acute disease. The inclusion criteria were patients of 65 years or older who were expected to be hospitalized for at least seven days, ability to cooperate and written informed consent. Patients with suspected or diagnosed dysphagia, paralysis or severe cognitive impairment (Montreal Cognitive Assessment (MoCA) < 10) [21] were excluded due to the impact on self-feeding or reporting appetite.

### 2.2. Geriatric Assessment

Barthel Index (BI) [22], FRAIL scale [23] and SARC-F questionnaire [24] were used to determine self-caring ability and functional status, respectively. Medical comorbidities and depressive symptoms were measured with the Charlson Comorbidity Index (CCI) [25] and the Depression in Old Age Scale (DIA-S) [26], respectively. The Mini Nutritional Assessment Short Form (MNA-SF) [27] and the Montreal Cognitive Assessment (MoCA) [21] were carried out in order to determine the nutritional status and cognitive function, respectively. Further, Edmonton Symptom Assessment System (ESAS) [28] and Simplified Nutritional Appetite Questionnaire (SNAQ) [29] were used to evaluate appetite, whereas the semi-quantitative plate diagram method was used to determine food intake [30].

### 2.3. Measurement of Inflammation

C-reactive protein (CRP), interleukin 1 beta (IL-1β), interferon alpha-2 (IFN-α2), interferon gamma (IFN-γ), tumor necrosis factor alpha (TNF-α), monocyte chemoattractant protein 1 (MCP-1), IL-6, IL-8, IL-10, IL-12p70, IL-17A, IL-18, IL-23 and IL-33 were measured. Briefly, serum-CRP was analyzed according to standard procedures and levels >3.0 (mg/dL) were considered as moderate to severe inflammation [31]. The immunoassay Human Inflammation Panel 1 LEGENDplexTM, Multi-Analyte Flow Assay Kit from BioLegend (San Diego, CA, USA) was used to measure serum-cytokine concentrations with the following reference range in healthy subjects according to the manufacturer’s kit manual: IL-1β, not detectable (ND)–20.4 pg/mL; IFN-α2, ND–155.8 pg/mL; IFN-γ, ND–15.0 pg/mL; TNF-α, ND–27.0 pg/mL; MCP-1, 72.7–431.5 pg/mL; IL-6, ND–13.1 pg/mL; IL-8, 3.4–77.1 pg/mL; IL-10, ND–9.9 pg/mL; IL-12p70, ND–4.7 pg/mL; IL-18, 53.7–276.9 pg/mL; IL-23, ND–94.2 pg/mL and IL-33, ND-192.2 pg/mL. The reference limit for IL-17A was ND–1.93 pg/mL [32].

All the measurements, except serum-cytokines, were performed within 24 h after hospital admission (baseline). Moreover, patients were assessed for food intake, appetite, CRP and cytokines at two occasions after their admission, i.e., at the time of the hospital admission (baseline) and at day 7 after the first assessment (follow-up). Serum samples for cytokine measurements were immediately frozen at −80 °C and analyzed collectively at the end of the study.

### 2.4. Data Analysis

The statistical analysis was performed using SPSS statistical software (SPSS Statistics for Windows, IBM Corp, Version 27, Armonk, NY, USA). Where appropriate, all data are reported as means and standard deviations (SDs), medians with interquartile ranges (IQR) and absolute numbers and percentages (*n*, %). Paired samples *t*-test and Wilcoxon signed rank were used to determine the differences in variables between baseline and follow-up. Moreover, the Spearman’s rank correlation coefficient was performed to test the associations between SNAQ-appetite and ESAS-appetite scores. The differences in cytokines changes and changes in appetite scores and food intake were explored using Kruskal–Wallis test, followed by pairwise comparison and one-way ANOVA with post hoc Tukey, respectively. Further, a stepwise multiple regression analysis was performed to investigate the independent effects of changes in cytokines and CRP levels, age, sex, infection and comorbidities on changes in patients’ appetite as dependent variables. A *p*-value of <0.05 was considered as the limit of significance.

## 3. Results

### 3.1. Characteristics of Study Population

The baseline characteristics of the study population can be seen in Table 1. Briefly, the age range was between 65 and 98 years (64% women). At baseline, 15 and 11% of the patients had a history of acute infections and a chronic inflammatory disease, respectively. In addition, 81% of the patients were frail, 92% had an impaired cognitive function, 42% exhibited depressive symptoms and 82% had probable sarcopenia according to SARC-F. At baseline, 47% (*n* = 89) of the patients had food intake <75% of the meals offered and 22% (*n* = 42) and 31% (*n* = 60) reported poor and very poor appetite according to ESAS and SNAQ, respectively. At follow-up, food intake, ESAS-appetite and SNAQ-appetite increased in 36, 37 and 32% of the patients, respectively (Table 1). In addition, significant negative correlations between the ESAS-appetite scores and the SNAQ-appetite scores at baseline (r = −0.661, *p* < 0.001) and between the changes at follow-up (r = −0.472, *p* < 0.001) were observed.

### 3.2. Changes in Inflammation Overtime

Table 2 shows the changes in CRP and cytokines levels from baseline to follow-up stratified by ESAS-appetite changes. At baseline, 30% (*n* = 58) of the total population had a CRP >3.0 (mg/dL). In addition, the mean serum concentrations of most cytokines were within the normal range on admission except MCP-1, IL-6, IL-8 and IL-18, which were elevated in 39, 65, 41 and 44% of the patients, respectively.

At the follow-up, the mean serum CRP levels significantly decreased in patients with an increasing appetite (*p* = 0.030) and remained unchanged overtime among the patients with a decreasing appetite (*p* = 0.586) or no change in appetite (*p* = 0.157). Further, no significant differences in the mean serum levels of all cytokines could be detected overtime, except for IL-18, which significantly increased in patients with a decreasing appetite (*p* = 0.001). Using SNAQ-appetite, only the mean serum IL-18 levels significantly increased in the group of patients with a decreasing appetite (320.0 ± 280.9 pg/mL at baseline vs. 372.5 ± 344.7 pg/mL at follow-up, *p* = 0.007).

### 3.3. Impact of Cytokines Changes on Appetite and Food Intake

**Figure 1:**
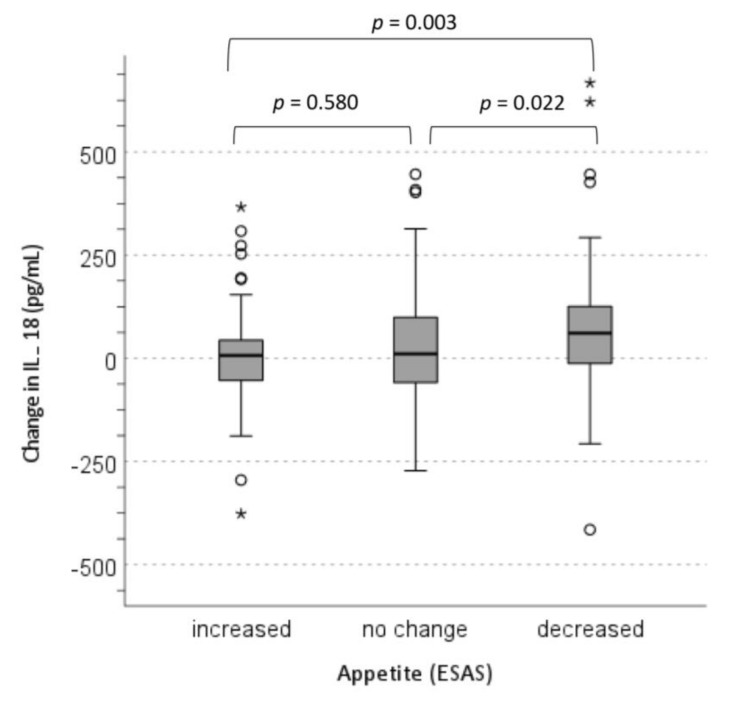
Comparison of changes in IL-18 levels across the ESAS-appetite levels in total population (*n*= 191). ESAS, Edmonton Symptom Assessment System; IL-18, interleukin 18. The circles (small values) and stars (extreme values) indicate the outliers.

A comparison of cytokines changes across the ESAS-appetite changes and food intake changes in the total population are shown in Table 3. There were significant differences in the changes in the mean concentrations of IL-6 (*p* = 0.018) and IL-18 (*p* = 0.009) with the changes in ESAS-appetite levels. For example, appetite significantly improved from baseline to follow-up in the patients with no significant change in IL-18 levels (Figure 1, mean changes in serum IL-18: 5.3 ± 121.1 pg/mL) and deteriorated in those with an increase in IL-18 levels (mean changes in serum IL-18: 78.6 ± 176.3 pg/mL; Figure 1). Even after excluding the patients with either an acute infection or chronic inflammatory diseases from the analysis, we observed similar findings concerning changes in ESAS-appetite levels and changes in IL-6 and IL-18 levels with the corresponding significance as *p* = 0.043 and *p* = 0.011, respectively.

Using SNAQ-appetite, significant differences between the changes in the mean concentrations of IL-10 (*p* = 0.021) and IL-33 (*p* = 0.012) with the changes in appetite levels were observed. Further, the changes in cytokines levels did not show any significant effect on food intake, expect for IL-18 (*p* = 0.002, Table 3). Indeed, food intake significantly increased in patients with a reduction in IL-18 levels (mean changes in serum IL-18 with an increasing food intake: −21.5 ± 174.1 pg/mL vs. in a decreasing food intake: 73.9 ± 147.0 pg/mL; *p* = 0.002).

In a stepwise regression analysis (Table 4), changes of IL-18 levels were the major independent predictor for changes of patients’ appetite (*p* = 0.014) and explained 4% of the variance, whereas other cytokines and variables such as age, sex, infection and disease did not show any impact on appetite changes. Using SNAQ-appetite, age (*p* = 0.010) was the only significant risk factor for changes of patients’ appetite.

## 4. Discussion

Previous studies have demonstrated significant associations between inflammatory biomarkers such as cytokines and appetite during inflammatory diseases. Indeed, the accumulation of cytokines, which are the key mediators of inflammation, may affect the satiety center in the hypothalamus, resulting in feeding suppression and diminished appetite [13,16,33]. To the extent of our knowledge, this is the first work assessing the effect of cytokine changes on food intake and appetite changes in older hospitalized patients. Our findings demonstrated that alterations of some biomarkers of the systemic inflammatory response such as IL-6 and IL-18 had a potential impact on appetite and food intake, whereas others had not. Our results revealed that the appetite of older hospitalized patients significantly deteriorated overtime in patients with increasing IL-18 levels and improved in those without significant changes in IL-18 levels.

Up to now, the knowledge regarding the effect of inflammation in the development of malnutrition is limited and the data are largely confined to cross-sectional studies and food intake, not appetite. It has been shown that cytokines, especially IL-6, IL-18 and TNFα, are involved in both inflammaging and different chronic conditions such as heart disease, kidney disease and cancer [16,17,34]. To illustrate, in a recent study among 76 older individuals (mean age 71 years), Fatyga et al. [34] demonstrated that inflammation is associated with malnutrition, irrespective of the etiology. In this study, the risk of malnutrition is positively and negatively associated with IL-8 and IL-18 levels, respectively [34].

In the present study, among all cytokines, changes in IL-18 levels overtime were the most evident independent predictor for changes in appetite and explained 4% of the variance compared to other risk factors. In addition, no other significant associations between changes in other cytokines and changes in appetite could be detected in the regression analysis. Of interest, the impact of IL-18 level changes on appetite changes was independent from the CRP level and infections. Our data suggest that changes in IL-18 levels were the main inflammatory mediator of appetite changes. Although there are very few studies on IL-18 as an anorectic inflammatory marker in humans, its regulative role as a potent anorectic cytokine was confirmed in animal studies. As an example, mice with an IL-18 deficiency reduced their food intake and lost weight when subjected to an intraperitoneal or intracerebroventricular injection of IL-18 [35]. In addition, similar animal studies reported significant associations between elevated levels of IL-18 and low food intake, which was consistent with the anorectic property of IL-18 [35,36,37]. Altogether, it appears that IL-18 plays a regulatory role in energy intake and energy homeostasis in diseased subjects. According to the fact that malnutrition and a loss of appetite in older subjects are mostly multifactorial, it is not surprising that IL-18 accounts for only 4% of the variance of appetite.

IL-18, which is an immunoregulatory cytokine, is a member of the IL-1 family and its serum levels are significantly elevated in a state of infection [35,37]. The primary sources of IL-18 are macrophages, dendritic cells and many other cells [38,39]. In addition to it acting in both acquired and innate immunity [40], IL-18 is also involved in chronic inflammation, autoimmune diseases and several cancers [41]. Without proof of causality, the findings of the present study suggest that IL-18 may exert a role in appetite regulation and mediate nutritional alterations during inflammatory conditions in diseased subject.

The measurement of appetite, which is a subjective sensory experience, is challenging [42]. In this study, appetite was evaluated using a single question of the SNAQ and one analogue scale of the ESAS. Although significant correlations between both tools at baseline and at follow-up were observed, none of the cytokine changes showed significant impact on SNAQ-appetite changes in the regression analysis. This discrepancy could be due to the fact that the SNAQ appetite question offers 5 possible answers, whereas the ESAS appetite score can differentiate between 11 different values. This may be the reason why the ESAS appetite assessment appears to be more effective in detecting changes of appetite.

Several limitations of this study should be discussed. Investigating a very heterogeneous group of older hospitalized patients may have influenced our findings, because there have been multiple reasons for a reduction in appetite in this cohort. Therefore, residual, uncontrolled confounding cannot be excluded. Notwithstanding, the relationship of the concurrent alterations of inflammatory biomarkers such as cytokines and appetite cannot prove but may substantiate causality. Future studies are required to reproduce the proposed anorexigenic effect of IL-18 in a population where inflammation may be the main cause of a loss of appetite.

## 5. Conclusions

We conclude that IL-18 seems to exert a significant impact on appetite in acutely ill older hospitalized patients and should, therefore, be considered as a potential target in the diagnosis, prevention and treatment of malnutrition.

## Figures and Tables

**Table 1:** Characteristics of the study population.

	Total Population (*n* = 191)
Gender	
Female (*n*; %)	122 (64)
Male (*n*; %)	69 (36)
Age (y)	81.3 ± 6.6
Height (m)	1.66 ± 0.08
Actual body weight (kg)	73.4 ± 18.0
BMI (kg/m^2^)	26.6 ± 6.2
MNA-SF, Median (IQR)	9 (7–10)
Normal nutritional status (*n*; %)	17 (9)
At risk of malnutrition (*n*; %)	113 (60)
Malnourished (*n*; %)	58 (31)
Barthel Index on admission, Median (IQR)	45 (35–55)
Frail scale, Median (IQR)	4 (3–4)
SARC-F, Median (IQR)	7 (4–8)
Cognitive function (MoCA), Median (IQR)	20 (15–23)
Depression score (DIA-S), Median (IQR)	3 (1–5)
Charlson Comorbidity Index, Median (IQR)	3 (2–4)
Infection on admission	
Yes (*n*; %)	29 (15)
No (*n*; %)	162 (85)
* Food intake at follow-up	
Increased (*n*; %)	66 (36)
No change (*n*; %)	45 (24)
Decreased (*n*; %)	75 (40)
ESAS-Appetite at follow-up	
Increased (*n*; %)	71 (37)
No change (*n*; %)	61 (32)
Decreased (*n*; %)	59 (31)
SNAQ-Appetite at follow-up	
Increased (*n*; %)	61 (32)
No change (*n*; %)	87 (46)
Decreased (*n*; %)	43 (22)

BMI, body mass index; MNA-SF, Mini Nutritional Assessment Short Form; MoCA, Montreal Cognitive Assessment; DIA-S, Depression in Old Age Scale; ESAS, Edmonton Symptom Assessment System; SNAQ, Simplified Nutritional Appetite Questionnaire. * Food intake was measured according to the plate diagram. Values are given as mean ± SD, number (%) or median (IQR, interquartile range).

**Table 2:** Changes in CRP and cytokines from baseline to follow-up stratified by ESAS-appetite status.

Decrease in ESAS-Appetite (*n* = 59)	Baseline	Follow-Up	Changes	*p* Value
CRP (mg/dL)	3.6 (6.0)	3.2 (5.5)	−0.3 (4.6)	0.586
IL-1β (pg/mL)	4.2 (5.9)	4.8 (7.1)	0.6 (4.9)	0.333
IFN-α2 (pg/mL)	5.0 (13.1)	5.0 (9.7)	0.0 (5.6)	0.992
IFN-γ (pg/mL)	4.3 (6.6)	4.7 (7.9)	0.4 (3.8)	0.466
TNF-α (pg/mL)	8.1 (10.9)	7.0 (10.7)	−1.1 (10.0)	0.412
MCP-1 (pg/mL)	355.9 (215.5)	382.9 (241.6)	27.0 (213.1)	0.334
IL-6 (pg/mL)	40.5 (49.9)	46.8 (58.1)	6.3 (59.9)	0.425
IL-8 (pg/mL)	104.3 (126.9)	114.7 (188.4)	10.4 (96.6)	0.413
IL-10 (pg/mL)	32.9 (121.8)	30.5 (107.4)	−2.4 (49.5)	0.707
IL-12p70 (pg/mL)	4.8 (6.8)	4.8 (6.9)	0.0 (2.7)	0.888
IL-17A (pg/mL)	3.0 (5.8)	3.7 (7.6)	0.7 (4.8)	0.255
IL-18 (pg/mL)	340.7 (268.3)	419.4 (333.5)	78.6 (176.3)	0.001
IL-23 (pg/mL)	7.6 (13.0)	8.0 (14.5)	0.4 (8.9)	0.698
IL-33 (pg/mL)	131.6 (249.1)	123.7 (250.1)	−7.8 (53.6)	0.267
Increase in ESAS-appetite (*n* = 71)				
CRP (mg/dL)	4.2 (6.9)	2.7 (3.9)	−1.5 (5.8)	0.030
IL-1β (pg/mL)	6.7 (10.7)	6.5 (8.9)	−0.2 (7.1)	0.816
IFN-α2 (pg/mL)	4.4 (6.5)	4.7 (6.3)	0.3 (3.3)	0.530
IFN-γ (pg/mL)	5.7 (7.8)	5.8 (7.5)	0.1 (4.5)	0.877
TNF-α (pg/mL)	8.4 (14.0)	6.5 (12.7)	−1.9 (8.4)	0.056
MCP-1 (pg/mL)	464.9 (328.0)	531.5 (589.9)	66.6 (461.6)	0.228
IL-6 (pg/mL)	55.3 (82.5)	45.8 (104.2)	−9.5 (124.5)	0.523
IL-8 (pg/mL)	109.9 (210.5)	114.6 (203.2)	4.7 (77.4)	0.608
IL-10 (pg/mL)	28.0 (141.8)	25.4 (115.3)	−2.6 (32.2)	0.497
IL-12p70 (pg/mL)	6.8 (9.0)	7.2 (11.5)	0.4 (8.6)	0.743
IL-17A (pg/mL)	3.6 (7.6)	2.8 (3.9)	−0.8 (5.7)	0.243
IL-18 (pg/mL)	313.4 (270.8)	318.7 (280.6)	5.3 (121.1)	0.714
IL-23 (pg/mL)	13.8 (26.8)	13.3 (27.8)	−0.4 (19.1)	0.848
IL-33 (pg/mL)	127.8 (187.6)	131.7 (197.8)	3.9 (105.6)	0.757
No change in ESAS-appetite (*n* = 61)				
CRP (mg/dL)	2.6 (3.8)	2.1 (2.7)	−0.5 (2.8)	0.157
IL-1β (pg/mL)	6.6 (8.7)	5.6 (11.9)	−1.0 (11.3)	0.506
IFN-α2 (pg/mL)	4.7 (7.8)	5.4 (13.0)	0.7 (7.9)	0.447
IFN-γ (pg/mL)	5.7 (9.5)	5.9 (8.4)	0.2 (4.3)	0.668
TNF-α (pg/mL)	9.9 (32.6)	13.6 (54.7)	3.6 (25.0)	0.263
MCP-1 (pg/mL)	414.1 (252.3)	451.0 (303.4)	36.9 (301.4)	0.343
IL-6 (pg/mL)	63.1 (132.9)	64.0 (142.4)	0.9 (146.7)	0.959
IL-8 (pg/mL)	93.6 (94.9)	99.6 (99.4)	6.0 (85.0)	0.582
IL-10 (pg/mL)	11.5 (26.9)	14.8 (36.6)	3.3 (24.3)	0.287
IL-12p70 (pg/mL)	5.4 (7.9)	5.2 (7.0)	−0.3 (3.6)	0.664
IL-17A (pg/mL)	2.5 (3.3)	2.4 (3.6)	−0.1 (2.1)	0.700
IL-18 (pg/mL)	328.6 (284.2)	341.6 (308.3)	13.0 (189.4)	0.594
IL-23 (pg/mL)	12.1 (28.4)	10.1 (23.0)	−2.0 (10.0)	0.122
IL-33 (pg/mL)	119.7 (171.4)	121.3 (170.5)	1.7 (76.0)	0.864

ESAS, Edmonton Symptom Assessment System; CRP, C-reactive protein; IL, interleukin; IFN-α2, interferon alpha-2; IFN-γ, interferon gamma; TNF-α, tumor necrosis factor alpha; MCP-1, monocyte chemoattractant protein 1. Values are given as mean (SD).

**Table 3:** Comparison of cytokines changes across the ESAS-appetite changes and food intake changes in total population (*n* = 191).

	Change in ESAS-Appetite Levels	Change in Food Intake *
Changes in Cytokines	*p* Value	*p* Value
IL-1β	0.098	0.489
IFN-α2	0.856	0.075
IFN-γ	0.862	0.053
TNF-α	0.058	0.498
MCP-1	0.925	0.106
IL-6	0.018	0.807
IL-8	0.947	0.512
IL-10	0.525	0.106
IL-12p70	0.876	0.900
IL-17A	0.604	0.108
IL-18	0.009	0.002
IL-23	0.662	0.956
IL-33	0.465	0.549

ESAS, Edmonton Symptom Assessment System; IL, interleukin; IFN-α2, interferon alpha-2; IFN-γ, interferon gamma; TNF-α, tumor necrosis factor alpha; MCP-1, monocyte chemoattractant protein 1. Differences of changes in cytokines and changes in appetite scores and food intake were performed using Kruskal–Wallis test and one-way ANOVA with post hoc Tukey, respectively. * Food intake was measured according to the plate diagram.

**Table 4:** Stepwise multiple regression analysis of risk factors associated with changes in ESAS-appetite in total population (*n* = 191).

	Changes in ESAS-Appetite		
	*Beta*	*t*	*p* Value
Changes in IL-1β	0.036	0.495	0.621
Changes in IFN-α	−0.007	−0.097	0.923
Changes in IFN-γ	0.004	0.056	0.955
Changes in TNF-α	0.034	0.461	0.645
Changes in MCP-1	−0.068	−0.926	0.356
Changes in IL-6	0.034	0.466	0.642
Changes in IL-8	−0.028	−0.363	0.717
Changes in IL-10	−0.014	−0.189	0.851
Changes in IL-12p70	−0.013	−0.177	0.860
Changes in IL-17A	0.131	1.804	0.073
Changes in IL-18	0.180	2.247	0.014
Changes in IL-23	0.023	0.316	0.753
Changes in IL-33	−0.039	−0.537	0.592
Changes in CRP	0.051	0.649	0.488
Age	0.121	1.673	0.741
Sex	−0.024	−0.331	0.096
Infection	−0.075	−1.022	0.308
CCI	0.046	0.628	0.531

ESAS, Edmonton Symptom Assessment System; IL, interleukin; IFN-α2, interferon alpha-2; IFN-γ, interferon gamma; TNF-α, tumor necrosis factor alpha; MCP-1, monocyte chemoattractant protein 1; CRP, C-reactive protein; CCI, Charlson Comorbidity Index.

## Data Availability

The datasets analyzed during the current study are available from the corresponding author on reasonable request.
